# LncRNA LHFPL3-AS1 contributes to tumorigenesis of melanoma stem cells via the miR-181a-5p/BCL2 pathway

**DOI:** 10.1038/s41419-020-03141-1

**Published:** 2020-11-04

**Authors:** Song Zhang, Haitao Wan, Xiaobo Zhang

**Affiliations:** College of Life Sciences and Laboratory for Marine Biology and Biotechnology of Qingdao National Laboratory for Marine Science and Technology, Zhejiang University, Hangzhou, 310058 The People’s Republic of China

**Keywords:** Cancer stem cells, RNA splicing

## Abstract

Long noncoding RNAs (lncRNAs) are recognized as a new area for cancer therapy. B-cell lymphoma-2 (Bcl-2)-mediated suppression of apoptosis is an important molecular hallmark of cancer. However, the influence of lncRNA on the regulation of oncogenic *Bcl-2* in cancer stem cells has not been explored. In this study, our findings revealed that the lncRNA LHFPL3-AS1-long, generated from the polypyrimidine tract binding protein 1 (PTBP1)-mediated splicing of the LHFPL3-AS1 precursor, upregulated BCL2 protein to contribute to tumorigenesis of melanoma stem cells. The in vitro and in vivo results showed that LHFPL3-AS1-long directly interacted with miR-181a-5p to inhibit the mRNA degradation of *Bcl-2* (the target of miR-181), thus suppressing apoptosis of melanoma stem cells. The splicing factor PTBP1 regulated the alternative splicing of LHFPL3-AS1 transcript by preferentially binding to the motifs located in exon3 of LHFPL3-AS1 precursor, leading to the biogenesis of LHFPL3-AS1-long in melanoma stem cells. In patients with melanoma, the expressions of PTBP1 and LHFPL3-AS1 were significantly upregulated compared with the healthy donors. Therefore, our study revealed a mechanistic crosstalk among an onco-splicing factor, lncRNA and tumorigenesis of melanoma stem cells, enabling PTBP1 and LHFPL3-AS1 to serve as the attractive therapeutic targets for melanoma.

## Introduction

Over the last few decades, the survival of patients with cancer has improved significantly and the combined cancer death rate has declined, primarily because of multidisciplinary care, improved chemotherapeutic agents and the incorporation of palliative care services into the management scheme^1^. However, a significant proportion of patients continue to experience recurrence of cancers after adjuvant treatment, and survival associated with stage IV solid tumors still remains low^2,3^. It is believed that the acquired resistance to chemotherapeutic agents and intratumoral heterogeneity are responsible for the failure of many of the agents used to treat patients with a malignancy. Recent studies have suggested that tumor heterogeneity is a result of the hierarchical organization of tumor cells by a subset of cells with stem cell features known as cancer stem cells^4^. These cancer stem cells display the capacity to self-renew, differentiate, and give rise to a new tumor, accounting for the high rate of cancerous recurrence^5^. Cancer stem cells often reprogram the expression profiles and levels of a wide range of genes to support their self-renewal and differentiation, which have been considered as hallmarks of stem cells. As well-known, microRNAs (miRNAs) are important post-transcriptional regulators of gene expression that act by direct base pairing to target sites within untranslated regions of mRNAs^6^. It has been found that miRNA activity can be affected by the presence of miRNA sponge transcripts, the so-called competing endogenous RNA (ceRNAs) in humans^7^. Multiple types of endogenous ceRNAs have been reported, including protein-coding transcripts, long noncoding RNAs (lncRNAs), pseudogenes, and circular RNAs (circRNAs)^7^. The conservation of ceRNA crosstalk in multiple organisms, including plants, zebrafish, mice, humans, and viruses, suggests that it may represent an important widespread layer of RNA regulation^8^. However, the regulatory principle of ceRNA in cancer stem cells is still unclear.

The complexity and diversity of potential ceRNA interactions have scaled exponentially with the identification of more than 10,000 lncRNAs^9^. Among the various types of ceRNAs, lncRNAs have been demonstrated as prominent competitive platforms for miRNAs^8^. Although the function and biological relevance of the vast majority of lncRNAs remain enigmatic, numerous lncRNAs have been reported to act as a prominent layer of transcriptional regulation in cancers^10^. THOR, a lncRNA characterized with ultraconserved vertebrate regions, represents a novel class of functionally important cancer and testis lncRNAs, which could regulate the expression of insulin growth factor 2^11^. Another lncRNA lncRNA-ATB activated by TGF-β has been reported to have the capability to induce epithelial–mesenchymal transition and to promote the colonization of disseminated tumor cells^12^. Recently, it is found that lncTCF7 is highly expressed in liver cancer stem cells through recruiting the SWI/SNF complex to the promoter of TCF7 (transcription factor 7) to regulate its expression, leading to the activation of Wnt signaling in hepatocellular cancer stem cells^13^. Although thousands of lncRNAs have been identified, the roles of lncRNAs in cancer stem cells have not been extensively investigated.

In order to elucidate whether and how the stemness of cancer stem cells was regulated by lncRNAs, the differentially expressed lncRNAs between melanoma stem cells and non-stem cells were analyzed in this study. The further analyses showed that LHFPL3-AS1 promoted tumorigenesis of melanoma stem cells by suppressing the Bcl-2 degradation.

## Materials and methods

### Cell culture

Melanoma stem cells and non-stem cells were sorted from MDA-MB-435 cells in our laboratory, which were performed as previously described^14^. Briefly, the top 1% ALDH1-positive cells were sorted from MDA-MB-435 cells with flow cytometry. As reported, one marker is not enough to fully characterize melanoma stem cells^15^. To confirm the sorted melanoma stem cells, the tumorsphere formation assay and tumorigenesis in nude mice of ALDH1-positive cells were conducted. All the data demonstrated that the sorted ALDH1-positive cells were melanoma stem cells^14^. Melanoma non-stem cells were cultured in Leibovitz’s L-15 medium (Sigma, USA) supplemented with 10% fetal bovine serum at 37 °C with 100% humidified atmosphere. Melanoma stem cells were grown in DMEM/F-12 medium (Invitrogen, USA) supplemented with 20 ng/mL epidermal growth factor (MedChemExpress, USA), 10 ng/mL basic fibroblast growth factor (MedChemExpress), 5 μg/mL of insulin (MedChemExpress), and 2% of B-27 (Invitrogen) at 37 °C in a humidified atmosphere with 5% CO_2_.

### RNA sequencing and differential expression analysis

Total RNAs were extracted from cells using Trizol (Invitrogen) according to the manufacturer’s protocol. The ribosomal RNA was removed using the Ribo-Zero™ kit (Epicentre, Madison, WI, USA). Fragmented RNAs, approximately 200 bp in length, were subjected to the first-strand and second strand cDNA synthesis, followed by adaptor ligation and enrichment with a low-cycle according to instructions of NEBNext^®^ Ultra™ RNA Library Prep Kit for Illumina (New England Biolabs Incorporation, USA). The purified library products were evaluated using the Agilent 2200 TapeStation and Qubit^®^2.0 (Life Technologies, USA). The libraries were paired-end sequenced by Guangzhou RiboBio Co., Ltd. (Guangzhou, China) using IlluminaHiSeq 3000 platform.

### Quantitative real-time PCR

Total RNAs were extracted from cells or tissues with an RNA isolation kit (Ambion, USA). The complementary DNA was synthesized with Reverse Transcription system (Toyobo, Osaka, Japan) according to the manufacturer’s instructions. Quantitative real-time polymerase chain reaction (PCR) was performed using SYBR Green PCR Master Mix (Vazyme Biotech Corporation, Nanjing, China) with sequence-specific primers (GAPDH, 5′-GGACCTGACCTGCCGTCTAG-3′ and 5′-GTAGCCCAGG ATGCCCTTGA-3′; LHFPL3-AS1-long, 5′-GTGAAGTCATGGGAAGGCAAG-3′ and 5′-CCCTCTCTGCCTGCAACTTAG-3′; LHFPL3-AS1-short, 5′-GCCTGATTGGTAGCCCTTGA-3′ and 5′-GTAGAGGAAAAGGCAGCCAGT-3′; ZFAS1, 5′-CAACTACTAGAGCGCCTCGG-3′ and 5′-CCAAAGATGGCTTTCGCACC-3′; LINC01531, 5′-TGGACCCTGTGCTTTGTCTC-3′ and 5′-GAGCACTGAACAACACTGCG-3′; U6, 5′-GTGCTCGCTTCGGCAGCACAT-3′ and 5′-ATGGAACGCTTCACGAATTT-3′; Oct4, 5′-CTGGGTTGATCCTCGGACCT-3′ and 5′-CCATCGGAGTTGCTCTCCA-3′; Sox2, 5′-GCCGAGTGGAAACTTTTGTCG-3′ and 5′-GGCAGCGTGTACTTATCCTTCT-3′; Nanog, 5′-TTTGTGGGCCTGAAGAAAACT-3′ and 5′-AGGGCTGTCCTGAATAAGCAG-3′; Klf4, 5′-ACCCTGGGTCTTGAGGAAGT-3′ and 5′-CTGATCGGGCAGGAAGGATG-3; PTBP1, 5′-TGACAAGAGCCGTGACTACAC-3′ and 5′-GCAGCTTGAGGAATGGCAAA-3′). Expression data were uniformly normalized to the internal control glyceraldehyde-3-phosphate dehydrogenase (GAPDH) and the relative expression levels were evaluated using the ΔΔCt method^16^.

### Isolation of nucleus and cytoplasm

Melanoma stem cells were washed twice with phosphate-buffered saline (PBS) and then suspended in 200 µl of buffer A [20 mM N-2-hydroxyethylpiperazine-N-ethane-sulphonicacid, 10 mM KCl, 1.5 mM MgCl_2_, 1 mM ethylene glycol bistetraacetic acid, 1 mM ethylene diamine tetraacetie acid, 1 mM dithiothreitol, 0.1 mM phenylmethylsulfonyl fluoride, 250 mM sucrose, pH 7.5] containing a protease inhibitor cocktail. The cells were homogenized and the homogenates were centrifuged twice at 750 × *g* for 5 min to collect nuclei. The resulting supernatant was centrifuged at 750 × *g* for 5 min to collect cytosolic fraction.

### Western blot

Proteins were separated by sodium dodecyl sulphate-polyacrylamide gel electrophoresis (SDS-PAGE) and then electrotransferred to a polyvinylidene fluoride membrane (Millipore, USA). After incubation in blocking solution (5% skim milk) for 2 h, the membrane was incubated with a primary antibody overnight at 4 °C, followed by incubation with alkaline phosphatase-conjugated secondary antibody (Roche, Switzerland) for 2 h at room temperature. The signal of the membrane was detected with 5-bromo-4-chloro-3-indolyl phosphate/nitro blue tetrazolium substrate (Sangon, China). The antibodies used in this study were purchased from Abcam (USA).

### Online data mining

Patients’ clinical profiles of PTBP1 and LHFPL3-AS1 and Kaplan–Meier survival analysis of clinical cases was obtained and analyzed using gene expression profiling interactive analysis (GEPIA) (http://gepia.cancer-pku.cn/index.html)^17^.

### Silencing of target gene expression by shRNA

To silence the expressions of target genes, shRNAs (LHFPL3-AS1-shRNA, 5′-GGACACCACTCAGGCTTATAA-3′, PTBP1-shRNA, 5′-CCCUCAUUGA CCUGCACAATT-3′) were designed by Vigene Bioscience Company (USA). As a control, the sequence of shRNAs were randomly scrambled, respectively. shRNAs were cloned into lentiviral vector pLent-U6-Puro (Vigene Bioscience, USA), followed by transfection into 293T cells using Lipofectamine 2000 reagent (Life Technologies, USA). At 48 h after transfection, the viral particles were collected to infect melanoma stem cells. Subsequently, the cells were cultured in medium contained 10 µg/ml puromycin for three days. After puromycin screening, only the cells with resistance were selected as stable strains expressing shRNA.

### Cell viability assay

Cell viability was monitored with MTS [3-(4, 5-dimethylthiazol-2-yl)-5-(3-carboxymethoxyphenyl)-2-(4-sulfophenyl)-2H-tetrazolium, inner salt] using a CellTiter 96^®^ AQueous One Solution Cell Proliferation Assay kit (Promega, USA) according to the manufacturer’s protocol. Briefly, 20 µl of CellTiter 96^®^ AQueous One Solution Reagent was added to the cells. Then the cells were incubated at 37 °C for 2 h. The absorbance was measured at 490 nm using the iMARKTM microplate reader (Bio-Rad, USA).

### Cell cycle assay

Fluorescence-activated cell sorting analysis was used to examine the cell cycle of melanoma stem cells. Cells were fixed in ice-cold ethanol overnight. Then the cells were treated with DNase-free RNase A (20 µg/mL) for 30 min. After centrifugation at 500 × *g* for 5 min, the cells were stained with propidium iodide (PI) (50 µg/mL). The fluorescence intensity of cells was measured with a flow cytometer at an excitation wavelength of 488 nm.

### Apoptosis detection

Cells were collected by centrifugation at 300 × *g* for 10 min. After washes with cold PBS, the cells were stained with fluorescein isothiocyanate (FITC)-Annexin V and PI using a FITC Annexin V apoptosis detection kit (BD Biosciences, USA) according to the manufacturer’s recommendations and then immediately analyzed by flow cytometry (BD Biosciences, USA). The percentage of apoptotic cells was calculated using Cell QuestPro software (BD Biosciences, USA).

### Tumorsphere formation assay

Tumorsphere formation assay was conducted under non-adherent and serum-free cell culture conditions. A single cell was plated into an ultra-low adherent 96-well plate and cultured in stem cell medium. After culture for 2 weeks, the cells were examined under a light microscope. The sphere-initiating cell frequency was calculated by using extreme limiting dilution analysis.

### RNA pulldown assay

The DNA sequence of LHFPL3-AS1-long or LHFPL3-AS1-short was amplified with sequence-specific primers containing T7 RNA polymerase promoter sequence. Then the purified PCR product was used as the template for in vitro transcription. The RNA transcript was synthesized using T7 in vitro transcription kit (Promega, USA) and biotinylated with EZ-Link Biotin kit (Thermo scientific, USA) according to the manufacturer’s instructions. The biotin-labeled RNAs were purified with mirVana miRNA Isolation Kit (Ambion, USA).

Cancer stem cells (5 × 10^6^) were lysed using immunoprecipitation lysis buffer (Beyotime, China) containing 2 mM protease inhibitor. After centrifugation at 300×*g* for 5 min, the cell lysate was incubated with the biotinylated sense or antisense LHFPL3-AS1 RNA at 4 °C overnight. Subsequently, the mixture was incubated with streptavidin-conjugated Dynabeads (Thermo Scientific, USA) on a rotator for 2 h at 4 °C. The beads were washed with lysis buffer and then boiled in protein loading buffer (Sangon Biotech, Shanghai, China). The proteins were separated by gel electrophoresis and stained with Coomassie brilliant blue (Beyotime, China). The separated proteins bands specific for the sense LHFPL3-AS1 RNA were excised for mass spectrometry analysis.

### Prediction of miRNAs targeted by lncRNA

The putative target miRNAs of LHFPL3-AS1-long were predicted using the miRanda, TargetScan, and PicTar algorithms. The overlapped targets of three algorithms were the potential targets of lncRNA.

### Dual-luciferase reporter assay

LHFPL3-AS1-long was amplified with sequence-specific primers (5′-TAAGAGCTCATCTAGGAACTGGAGCTGCT-3′ and 5′-AACGTCGACCCGCATTCAAAAACATCCTG-3′). As a control, the potential binding site of LHFPL3- AS1-long to miR-181 was mutated by PCR using sequence-specific primers (5′-ATGTAAGTATGCTGCACACCTTTCCCATCA-3′ and 5′-AGCATACTTACATACAGGCAGCCACACCTG-3′). The wild-type and mutant LHFPL3-AS1-long were cloned into the pmirGLO dual-luciferase miRNA target expression vector (Promega, USA). Subsequently 50 nM of the synthesized miR-181a-5p (5′-AACAUUCAACGCUGUCGGUGAGU-3′) or control miRNA (5′-AUCCUACGACAGUGCCGGAGAAU-3′) was co-transfected with 2 µg of the plasmid expressing wild-type or mutant LHFPL3-AS1-long into MDA-MB-435 cells using Lipofectamine 2000. At 36 h after transfection, the luciferase activity of cells was measured using the dual-luciferase reporter assay system (Promega, USA) according to the manufacturer’s protocol.

### Overexpression and rescue of gene expression

The sequence of a target gene was amplified using sequence-specific primers (PTBP1, 5′-GCAGAATTCATGGACGGCATTGTCCCAGATA-3′ and 5′-ATAGGATCCCTAGATGGTGGACTTGGAGAAG-3′; LHFPL3-AS1-long, 5′-AGTGAATTCATCTAGGAACTGGAGCTGCTGC-3′ and 5′-ACTGGATCCTCAAAAACATCCTGTCCGGTTG-3′; LHFPL3-AS1-short, 5′-AGTGAATTCATCTAGGAACTGGAGCTGCTGC-3′ and 5′-ACTGGATCCCCAAGTGACCTATGAGTAGAGGA-3′; LHFPL3-AS1-exon3, 5′-GCAGAATTCGTAAAAGCAAAAGGAAAATGAGG-3′ and 5′-ACTGGATCCTCAAAAACATCCTGTCCGGTTG-3′) and then cloned into the pCDH vector (Vigene Biosciences, USA). The control miRNA and miR-181a-5p were synthesized by GenePharma (Shanghai, China). Subsequently a recombinant plasmid or miRNA was transfected into cells using Lipofectamine 2000. As a control, vector alone was included in the transfection. At different time after transfection, the cells were collected for later use.

### Tumorigenicity in nude mice

Melanoma stem cells were transfected with LHFPL3-AS1-shRNA or PTBP1-shRNA to silence the expression of PTBP1 or LHFPL3-AS1. LHFPL3-AS1-shRNA-scrambled or PTBP1-shRNA-scrambled was included in the transfection as a control. Then the cells were collected at 6 × 10^6^ cells/mL in physiological saline. Matrigel (Becton, Dickinson and Company, USA) was added to the cell suspension at a ratio of 1:2. Subsequently, 250 µl of the cell suspension was subcutaneously injected into BALB/c nude mice to induce tumor growth. The tumor volume was examined weekly. Seven weeks later, the nude mice were sacrificed and their tumor sizes were evaluated. Animal experiments were approved by The Animal Experiment Center of Zhejiang University, China. All methods were carried out in accordance with approved guidelines.

### Immunohistochemical analysis

To examine proteins in solid tumors of mice by immunohistochemical staining, 5 μm-thick section was placed on a precoated slide with 3-triethoxysilylpropylamine (Merck, Darmstadt, Germany). The slide was soaked in xylol for 1 h and washed in series of decreasing alcohol concentrations. After deparaffinising the tissue, antigen retrieval of the section was performed in a microwave for 5 min in TEC buffer (0.05 M Tris-HCl, 0.05 M ethylenediaminetetraacetic acid, 0.02 M Na-Citrate, pH7.8), followed by peroxidase blocking. Then the slide was incubated with a primary antibody for 12 h and a subsequent incubation with the biotinylated secondary antibody (Vector, Grünberg, Germany) for 30 min. The slide was stained with diaminobenzidine (Sigma, USA) for 10 min to label proteins and then counterstained with haematoxylin to label nuclei.

### RNA interference (RNAi) assay

To silence the expression of Bcl-2 in melanoma stem cells, RNAi assay was conducted. The melanoma stem cells (1 × 10^5^) were transfected with 50 nM of siRNA with Lipofectamine 2000 (Invitrogen, USA). As a control, Bcl-2-siRNA-scrambled was included in the transfection. All the siRNAs were synthesized by GenePharma Co., Ltd. (Shanghai, China). At different time after transfection, the cells were harvested for later use.

### CLIP assay

Cells were irradiated in an ultraviolet (UV) cross-linker for 400 mJ/cm^2^ and then for 200 mJ/cm^2^. After centrifugation at 300×*g* for 5 min, the cells were resuspended in immunoprecipitation lysis buffer (Beyotime, China), followed by incubation with DNase at 37 °C for 20 min. The lysate was centrifuged at 10,000×g for 10 min to remove the cell debris and then precleared by incubation with Protein A Dynabeads (Life Technologies, USA) at 4 °C for 60 min. The supernatant was incubated with anti-PTBP1 IgG or mouse IgG (Beyotime, China) at 4 °C overnight, followed by incubation with Protein A Dynabeads at 4 °C for 2 h. After washes with PBST (0.5% Tween20 in PBS), the beads were subjected to 12% SDS-PAGE with Coomassie staining. At the same time, the RNAs in the precipitated complex were extracted using a mirVanaP miRNA isolation kit (Ambion Thermo, USA) and analyzed by quantitative real-time PCR.

### Semiquantitative RT-PCR

The total RNAs were extracted from the cells using an RNA isolation kit (Ambion, USA). Subsequently, the first-strand cDNA was synthesized by reverse transcription with a PrimeScript 1st strand cDNA synthesis kit (TaKaRa, Japan).The cDNA was used as the template for PCR amplification. The products of PCR were separated on a 1% agarose gel and stained with ethidium bromide. The primers used for amplification as follows: GAPDH, 5′-TCCATGGCACCGTCAAGGCT-3′, 5′-CACTGACACGTTGGCAGTGG-3′, LHFPL3-AS1-long, 5′-ATGGGAAGGCAAGTCATGAA-3′, 5′-GGCCCGAGCATCAGCCTGTG-3′.

## Results

### Upregulation of lncRNA LHFPL3-AS1 in melanoma stem cells

**Fig. 1:**
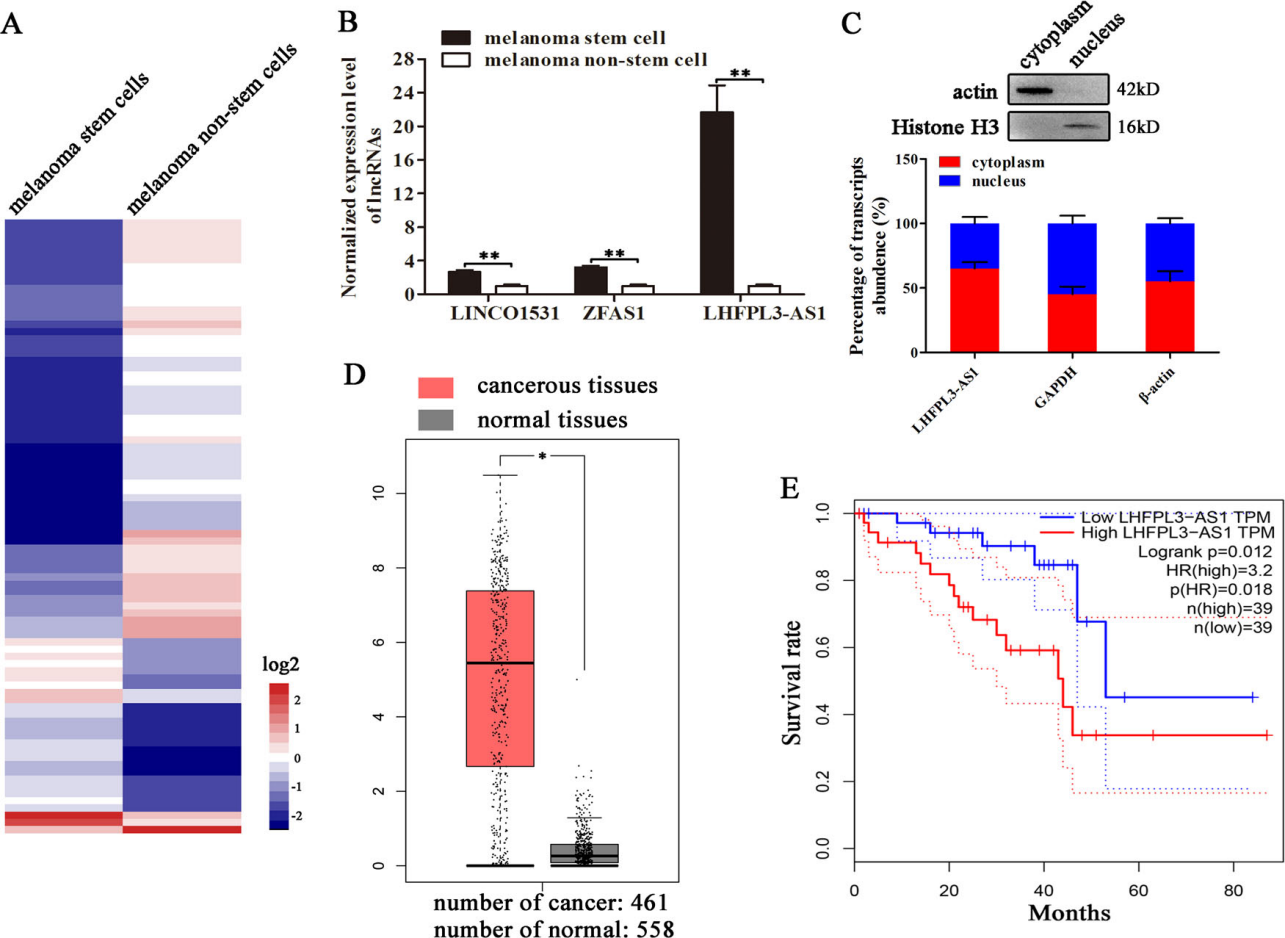
Upregulation of lncRNA LHFPL3-AS1 in melanoma stem cells. **A** The expression heatmap of lncRNAs in melanoma stem and non-stem cells. **B** The differential expressions of lncRNAs in melanoma stem and non-stem cells. The expression levels of lncRNAs were investigated using quantitative real-time PCR (**p < 0.01). **C** Expression of LHFPL3-AS1 in nucleus and cytoplasm of melanoma stem cells. The purity of nucleus and cytoplasm was determined by Western blot using actin-specific and histone H3-specific antibody, respectively (up). The expression level of LHFPL3-AS1 was evaluated with quantitative real-time PCR (down). **D** Expression level of LHFPL3-AS1 in cancerous and normal tissues of patients with melanoma. The expression profiles of LHFPL3-AS1 were evaluated using GEPIA database (*p < 0.05). **E** Curves for overall survival of melanoma patients with high vs. low expression of LHFPL3-AS1.

To explore the regulatory roles of lncRNAs in cancer stem cells, melanoma stem cells and non-stem cells were subjected to RNA sequencing and analysis. The results of differential expression analysis indicated that 85 lncRNAs were differentially expressed in melanoma stem cells compared with melanoma non-stem cells (Fig. 1A). To confirm the differential expression profiles of lncRNAs, three upregulated lncRNAs (LHFPL3-AS1, ZFAS1, and LINCO1531) were characterized by quantitative real-time PCR. The results showed that three selected lncRNAs were significantly upregulated in melanoma stem cells (Fig. 1B). Among the upregulated lncRNAs, LHFPL3-AS1 was the most highly expressed lncRNA in melanoma stem cells, suggesting its important role in melanoma stem cells. Thus, LHFPL3-AS1 was further characterized.

The results showed that LHFPL3-AS1 was expressed in both nucleus and cytoplasm (Fig. 1C), suggesting its complex function in melanoma stem cells. Based on GEPIA database^17^, it was found that LHFPL3-AS1 was significantly upregulated in cancerous tissues of patients with melanoma compared with the adjacent normal tissues (Fig. 1D). Kaplan–Meier survival analysis revealed that the patients with high LHFPL3-AS1 level melanomas had shorter survival rate compared with those with low LHFPL3-AS1 expression level (Fig. 1E). Collectively, these data demonstrated that LHFPL3-AS1 was associated with melanoma progression.

### Role of LHFPL3-AS1 in melanoma stem cells

**Fig. 2:**
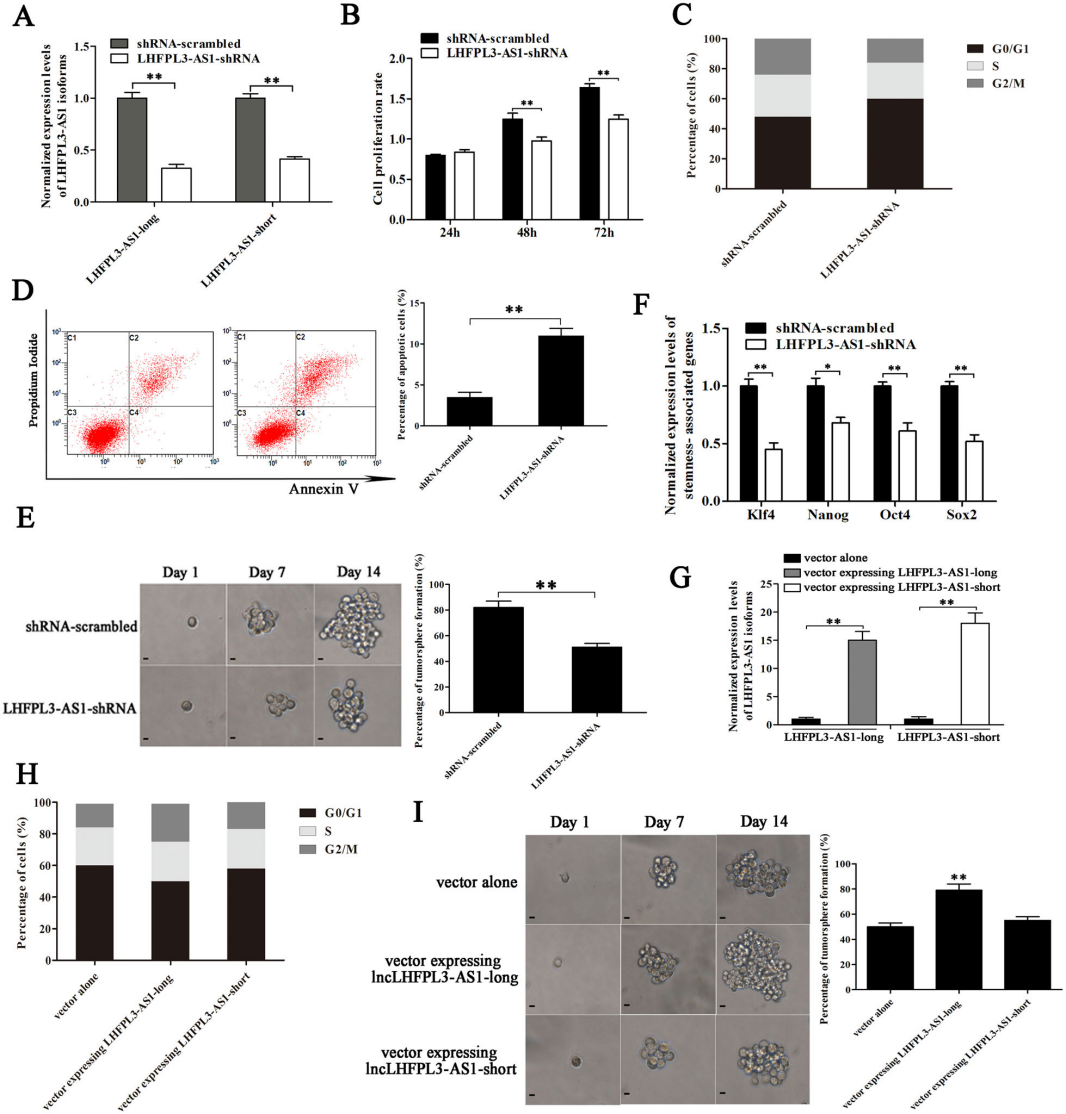
Role of HLFPL3-AS1 in melanoma stem cells. **A** Silencing of LHFPL3-AS1 expression in melanoma stem cells. LHFPL3-AS1-shRNA targeting LHFPL3-AS1 was transfected into melanoma stem cells to construct stable clones. As a control, shRNA-scrambled was used. The knockdown efficiency of LHFPL3-AS1 isoforms (LHFPL3-AS1-long and LHFPL3-AS1-short) was examined using quantitative real-time PCR (**p < 0.01). **B** Influence of LHFPL3-AS1 knockdown on melanoma stem cell proliferation. MTT assays were performed to examine the viability of melanoma stem cells (**p < 0.01). **C** Impact of LHFPL3-AS1 silencing on cell cycle. The cell cycle of melanoma stem cells with depleted LHFPL3-AS1 was analyzed using flow cytometry (**p < 0.01). **D** Effects of LHFPL3-AS1 depletion on cell apoptosis. Annexin V assays were performed to detect apoptosis of LHFPL3-AS1-silenced melanoma stem cells (**p < 0.01). **E** Impact of LHFPL3-AS1 knockdown on tumorsphere formation capacity of melanoma stem cells. The percentage of tumorsphere formation of melanoma stem cells and non-stem cells was evaluated (right). The representative images of tumorspheres were shown (left). Scale bar, 10 µm. **F** Downregulation of stemness-associated genes in LHFPL3-AS1-silenced melanoma stem cells. Quantitative real-time PCR was used to detect the expression levels of stemness-associated genes (*p < 0.05). GAPDH was used as normalization. **G** Expressions of LHFPL3-AS1 isoforms in melanoma stem cells. The cells were transfected with the vector expressing LHFPL3-AS1-long or LHFPL3-AS1-short. At 36 h after transfection, the expression levels of LHFPL3-AS1 isoforms were examined using quantitative real-time PCR (**p < 0.01). **H** Effects of LHFPL3-AS1 isoform rescue on cell cycle of the LHFPL3-AS1-knocked-down melanoma stem cells. LHFPL3-AS1-long or LHFPL3-AS1-short was overexpressed in LHFPL3-AS1 stably silenced cells. **I** Impact of LHFPL3-AS1 overexpression on tumorsphere formation capacity of melanoma stem cells. The melanoma stem cells were transfected with the vector expressing LHFPL3-AS1-long or LHFPL3-AS1-short. At day 14 after transfection, the percentage of tumorsphere formation of melanoma stem cells was evaluated (left). The representative images of tumorspheres were shown (right). Scale bar, 10 µm.

In order to characterize the role of LHFPL3-AS1 in melanoma stem cells, LHFPL3-AS1 was silenced using lentivirus-mediated short hairpin RNA (shRNA). LHFPL3-AS1 has two isoforms (National Center for Biotechnology Information accession no. 645591) the long variant (1334 bp) and the short variant (973 bp). The LHFPL3-AS1-shRNA could target both isoforms. Quantitative real-time PCR results showed that the expressions of both LHFPL3-AS1 isoforms were significantly knocked down by sequence-specific shRNA compared with the control (Fig. 2A). MTS assays revealed that the LHFPL3-AS1 silencing led to a significant decrease of melanoma stem cell proliferation rate (Fig. 2B). Compared with the control shRNA-scrambled, LHFPL3-AS1-silenced melanoma stem cells emerged significant cell cycle arrest in G0/G1 stage (Fig. 2C). The results of Annexin V assays showed that the LHFPL3-AS1 depletion induced apoptosis of melanoma stem cells (Fig. 2D). These data indicated that LHFPL3-AS1 depletion suppressed the proliferation of melanoma stem cells, leading to apoptosis of melanoma stem cells.

To further evaluate the influence of LHFPL3-AS1 silencing on the stemness of melanoma stem cells, tumorsphere forming capability and expression levels of stemness-associated genes of LHFPL3-AS1-silenced melanoma stem cells were investigated. The LHFPL3-AS1 depletion dramatically reduced tumorsphere forming capability of melanoma stem cells compared with the control (Fig. 2E). At the same time, the expression levels of stemness-associated genes were downregulated in LHFPL3-AS1-silenced melanoma stem cells (Fig. 2F). These data indicated that LHFPL3-AS1 played a positive role in the stemness of melanoma stem cells.

To explore which isoform of LHFPL3-AS1 was required for maintaining the stemness of melanoma stem cells, the expression of LHFPL3-AS1 isoforms was respectively overexpressed in the LHFPL3-AS1-knocked-down melanoma stem cells by transfecting the vector expressing LHFPL3-AS1-long or LHFPL3-AS1-short (Fig. 2G). The results indicated that the overexpression of LHFPL3-AS1-long rescued the cell cycle of the LHFPL3-AS1-knocked-down melanoma stem cells, while the cell cycle of the LHFPL3-AS1-short treatment was comparable to that of the control (Fig. 2H). At the same time, the tumorsphere formation capacity of the cells treated with LHFPL3-AS1-long was significantly increased compared with LHFPL3-AS1-short and the control (Fig. 2I). These data demonstrated that LHFPL3-AS1-long took positive effects on the stemness of melanoma stem cells.

Taken together, it could be concluded that LHFPL3-AS1-long isoform was required for maintaining the stemness of melanoma stem cells.

### Suppression of Bcl-2 degradation by LHFPL3-AS1-long in melanoma stem cells

**Fig. 3:**
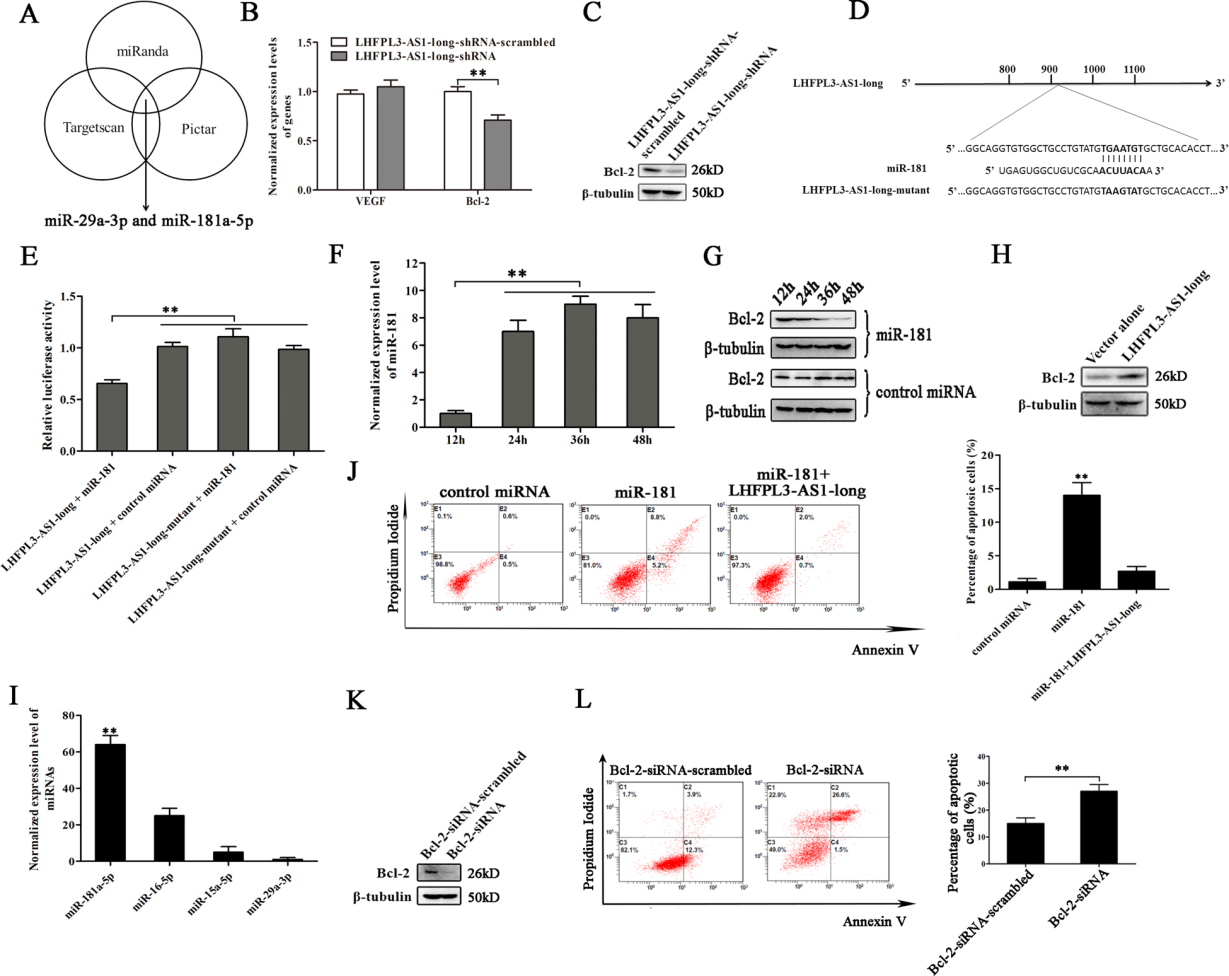
Suppression of Bcl-2 degradation by LHFPL3-AS1-long in melanoma stem cells. **A** The potential miRNAs targeted by LHFPL3-AS1-long. Three algorithums (miRanda, Targetscan, and Pictar) were used to predict the target miRNAs of LHFPL3-AS1-long. The overlapped miRNAs were predicted to be potential targets of LHFPL3-AS1-long. **B** Influence of LHFPL3-AS1-long silencing on the target genes’ expressions of miRNAs in melanoma stem cells. The LHFPL3-AS1-long-treated melanoma stem cells were subjected to quantitative real-time PCR to examine the expressions of miRNAs’ targets. **C** Western blot analysis of miRNAs’ targets in the LHFPL3-AS1-long-silenced melanoma stem cells. β-tubulin was used as a control. **D** Schematic diagram of the LHFPL3-AS1-long binding bases to miR-181. **E** Direct interaction between LHFPL3-AS1-long and miR-181. MDA-MB-435 cells were co-transfected with miR-181 and a luciferase reporter fused with LHFPL3-AS1-long. The firefly and renilla luciferase activities were analyzed. As controls, control miRNA and LHFPL3-AS1-long mutant were included in the co-transfections (**p < 0.01). **F** Overexpression of miR-181 in melanoma stem cells. Melanoma stem cells were transfected with the synthesized miR-181a-5p. At different time after transfection, the expression level of miR-181 was examined (**p < 0.01). **G** Impact of miR-181 overexpression on the expression of Bcl-2 in melanoma stem cells. β-tubulin was used as a loading control. **H** Effects of LHFPL3-AS1-long overexpression on the Bcl-2 expression in melanoma stem cells. Melanoma stem cells were transfected with LHFPL3-AS1-long and the expression level of Bcl-2 was examined using Western blot. **I** Expression levels of miRNAs targeting Bcl-2 in melanoma stem cells. The expressions of miRNAs were examined with quantitative real-time PCR (**p < 0.01). **J** Influence of miR-181 or LHFPL3-AS1-long overexpression on apoptosis of melanoma stem cells. The cells transfected with miR-181 or/and LHFPL3-AS1-long were subjected to apoptosis detection with flow cytometry (**p < 0.01). **K** Knockdown of Bcl-2 expression in melanoma stem cells. The expression of Bcl-2 in melanoma stem cells was silenced using sequence-specific siRNA. Melanoma stem cells were transfected with Bcl-2-siRNA. At 36 h after transfection, western blot was conducted to detect the protein level. β-tubulin was used as a control. **L** Effects of Bcl-2 silencing on apoptosis of melanoma stem cells (**p < 0.01).

To reveal the LHFPL3-AS1-mediated regulatory mechanism on the proliferation of melanoma stem cells, the miRNAs bound to LHFPL3-AS1-long were predicted. The prediction analysis showed that miR-29a-3p and miR-181a-5p (simplified as miR-29 and miR-181) were the potential targets of LHFPL3-AS1-long (Fig. 3A). As reported, *VEGF* and *Bcl-2* genes are the targets of miR-29 and miR-181, respectively^18,19^. Thus the expression levels of *VEGF* and *Bcl-2* genes were characterized in the LHFPL3-AS1-long-silenced melanoma stem cells. Quantitative real-time PCR and Western blot results indicated that the LHFPL3-AS1 knockdown led a significant decrease of Bcl-2 expression in melanoma stem cells, but had no effect on VEGF expression (Fig. 3B, C). Therefore, miR-181 was further characterized.

To evaluate the direct interaction between LHFPL3-AS1-long and miR-181, the dual-luciferase report plasmid expressing wild-type or mutant LHFPL3-AS1-long was constructed (Fig. 3D) and then co-transfected with miR-181 into MDA-MB-435 cells. The results showed that the luciferase activity of the cells co-transfected with miR-181 and LHFPL3-AS1-long was significantly decreased compared with the mutant LHFPL3-AS1-long (Fig. 3E), indicating that miR-181 directly interacted with LHFPL3-AS1-long. The above data manifested that LHFPL3-AS1 suppressed apoptosis of melanoma stem cells and Bcl-2 was a target gene of miR-181. As well-known, Bcl-2 plays an important role in apoptosis. In this context, the influence of the interactions between LHFPL3-AS1, miR-181 and Bcl-2 on apoptosis of melanoma stem cells was further evaluated.

To explore the miR-181-mediated pathway, miR-181 was overexpressed in melanoma stem cells (Fig. 3F). Western blot data demonstrated that the miR-181 overexpression significantly decreased the expression level of its target gene *Bcl-2* compared with the control (Fig. 3G). To examine the impact of LHFPL3-AS1 on the Bcl-2 expression, LHFPL3-AS1-long was overexpressed in melanoma stem cells. Western blot results indicated that the LHFPL3-AS1-long overexpression increased the BCL-2 expression in melanoma stem cells (Fig. 3H). Based the prediction of target genes, Bcl-2 could be targeted by miR-15a-5p/16-5p except for miR-181. The quantitative real-time PCR data showed that miR-181a-5p was highly expressed in melanoma stem cells compared with miR-15a-5p/16-5p (Fig. 3I), suggesting that miR-181 was important in melanoma stem cells. To further evaluate the impact of miR-181 and LHFPL3-AS1-long on apoptosis of melanoma stem cells, the cells were transfected with LHFPL3-AS1-long or/and miR-181, followed by apoptosis detection. Flow cytometry data showed that the miR-181 overexpression induced apoptosis of melanoma stem cells compared with the controls, while the LHFPL3-AS1-long overexpression inhibited miR-181-mediated apoptosis (Fig. 3J).

To characterize the role of Bcl-2 in melanoma stem cells, the expression of Bcl-2 was silenced using sequence-specific siRNA (Fig. 3K). The results showed that the Bcl-2 silencing significantly promoted apoptosis of melanoma stem cells compared with the control (Fig. 3L), indicating that Bcl-2 played a negative role in apoptosis of melanoma stem cells.

Collectively, these findings demonstrated that LHFPL3-AS1-long directly interacted with miR-181 to inhibit the mRNA degradation of *Bcl-2* (the target of miR-181), thus suppressing apoptosis of melanoma stem cells.

### Interaction between LHFPL3-AS1 and PTBP1 protein in melanoma stem cells

**Fig. 4:**
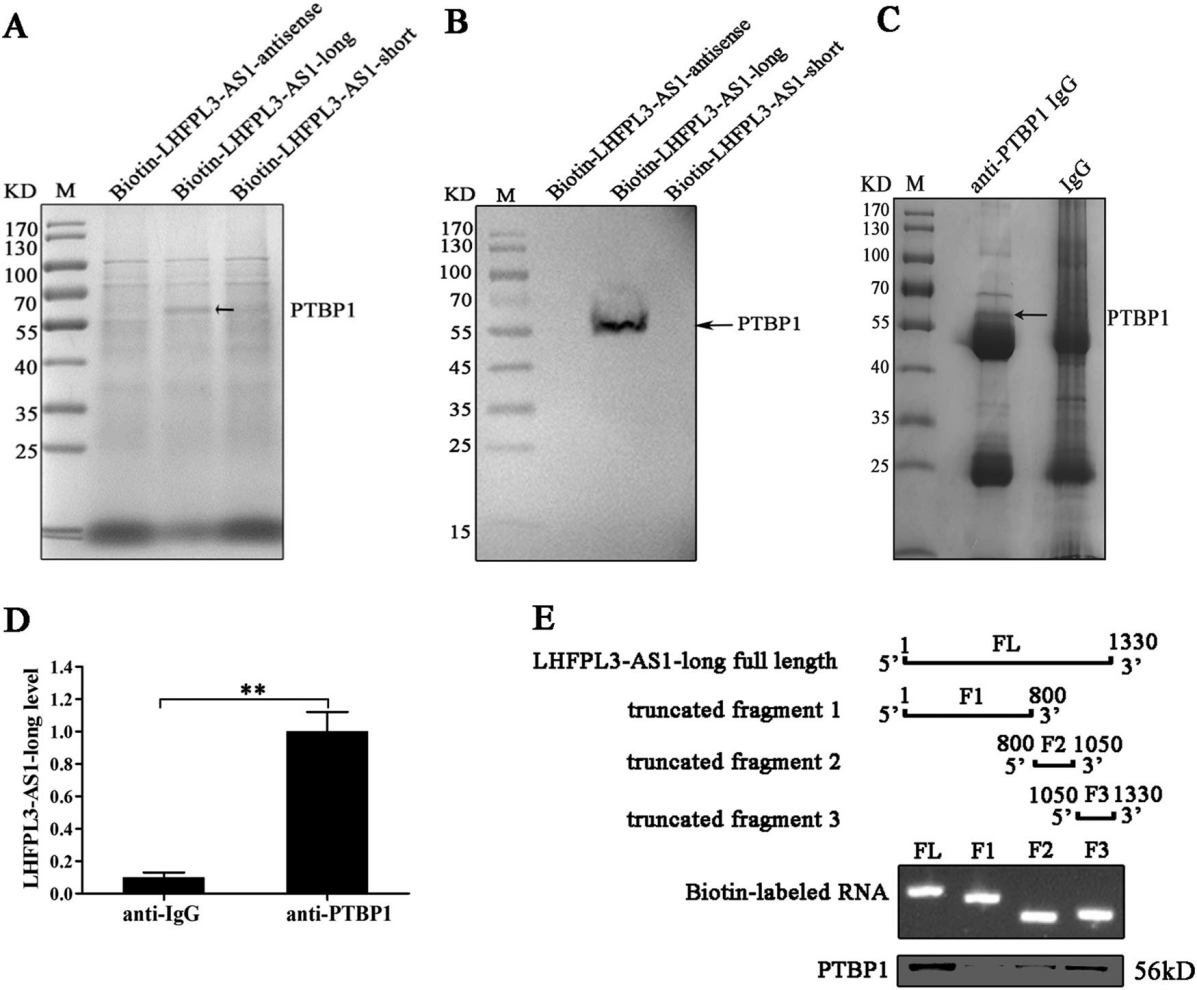
Interaction between LHFPL3-AS1 and PTBP1 protein in melanoma stem cells. **A** RNA pull-down assay. The cell lysate of melanoma stem cells sorted from MDA-MB-435 was incubated with the biotin-labeled sense or antisense LHFPL3-AS1. The proteins bound to LHFPL3-AS1 were separated by SDS-PAGE and stained with Coomassie brilliant blue and the differential protein was identified by mass spectrometry. The arrow indicated the identified protein. M, protein marker. **B** Western blot analysis of the protein bound to LHFPL3-AS1. The products of RNA pull-down assays were analyzed using Western blot with anti-PTBP1 antibody. The arrow showed the PTBP1 protein. **C** Interaction between LHFPL3-AS1-long and PTBP1 protein in melanoma stem cells. Cross-linking and immunoprecipitation (CLIP) assay using PTBP1-specific antibody or IgG was conducted in melanoma stem cells. The immunoprecipitated complex was examined by SDS-PAGE with Coommassie staining. M, protein marker. **D** LHFPL3-AS1-long detection in the immunoprecipitated complex. The LHFPL3-AS1-long level was determined with quantitative real-time PCR (**p < 0.01). **E** The LHFPL3-AS1-long region interacting with the PTBP1 protein. The full-length LHFPL3-AS1-long was truncated (top panel) and analyzed with 1% agarose gel electrophoresis (middle panel). Then the biotin-labeled fragments were incubated with the whole cell lysate, followed by the detection of PTBP1 protein binding using Western blot (bottom panel).

To reveal the LHFPL3-AS1-mediated regulatory mechanism on the proliferation of melanoma stem cells, the proteins binding to LHFPL3-AS1 were characterized. The RNA pull-down results using LHFPL3-AS1 RNA indicated that a specific protein band was observed for the LHFPL3-AS1-long compared with the control (Fig. 4A). Mass spectrometry analysis revealed that the protein was polypyrimidine tract binding protein 1 (PTBP1) (Fig. 4A). Western blotting with the anti-PTBP1 antibody confirmed the mass spectrometric data (Fig. 4B). To further confirm the interaction between LHFPL3-AS1-long and PTBP1 protein in melanoma stem cells, cross-linking and immunoprecipitation (CLIP) assay using the PTBP1-specific antibody was conducted. The results showed that the PTBP1 complex was precipitated (Fig. 4C). The level of LHFPL3-AS1-long was much higher in the immunoprecipitated PTBP1 complex than that in the control (Fig. 4D). These data demonstrated that LHFPL3-AS1-long was specifically interacted with the PTBP1 protein.

To explore the LHFPL3-AS1-long region interacting with the PTBP1 protein, a series of truncated fragments of LHFPL3-AS1 were constructed (Fig. 4E). The results of RNA pull-down assays revealed that a 250-bp fragment (the 1050th to the 1330th nucleotides) of LHFPL3-AS1-long was required for the interaction between LHFPL3-AS1-long and the PTBP1 protein (Fig. 4E). In this 250-bp fragment, there existed the known PTBP1 binding sites (UCUU and UCUCU)^20^.

Taken together, these findings revealed that LHFPL3-AS1-long was interacted with the PTBP1 protein.

### Role of PTBP1 in melanoma stem cells

**Fig. 5:**
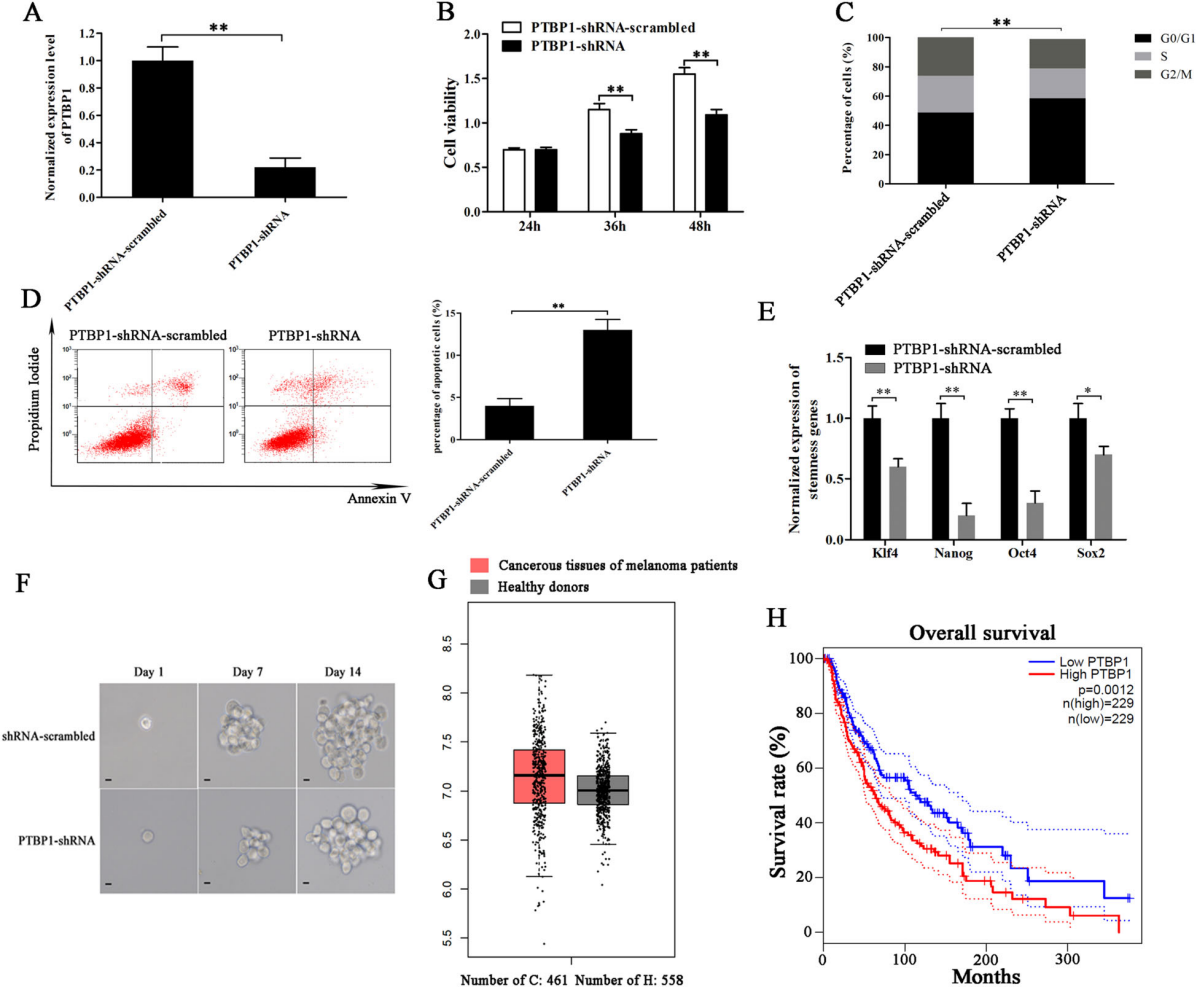
Role of PTBP1 in melanoma stem cells. **A** Knockdown of PTBP1 in melanoma stem cells. Melanoma stem cells stably expressing PTBP1 shRNA were established using lentivirus transfection and the expression level of PTBP1 was determined using quantitative real-time PCR (**p < 0.01). **B** Cell viability analysis. Melanoma stem cells were transfected with PTBP1-shRNA. At different time after transfection, the cells were subjected to cell viability analysis (**p < 0.01). **C** Cell cycle analysis. Flow cytometry analysis of melanoma stem cells treated with PTBP1-shRNA or PTBP1-shRNA-scrambled was conducted. The percentage of cells at different phases of cell cycle was indicated (**p < 0.01). **D** Role of PTBP1 in apoptosis of melanoma stem cells. Apoptosis of melanoma stem cells treated with PTBP1-shRNA or PTBP1-shRNA-scrambled was examined using flow cytometry (**p < 0.01). **E** Effects of PTBP1 silencing on the expressions of stemness genes in melanoma stem cells. The expressions of stemness genes in melanoma stem cells were examined with quantitative real-time PCR (**p < 0.01). **F** Tumorsphere formation ability investigation of melanoma stem cells treated with PTBP1-shRNA or PTBP1-shRNA-scrambled. **G** Expression level of PTBP1 in solid tumors of melanoma patients. Based on GEPIA database (http://gepia.cancer-pku.cn/index.html), the expression of PTBP1 in the solid tumors of patients with melanoma and healthy donors was evaluated. **H** Overall survival rate of melanoma patients with high vs. low expression of PTBP1.

In order to characterize the role of PTBP1 in melanoma stem cells, melanoma stem cells stably overexpressing PTBP1-targeted shRNA were established using lentivirus transfection. Quantitative real-time PCR showed that PTBP1 was knocked down in melanoma stem cells (Fig. 5A). The PTBP1 silencing led to a significant decrease of the viability of melanoma stem cells compared with the control (Fig. 5B), suggesting that PTBP1 had a positive role on the proliferation of melanoma stem cells. To further determine the effects of PTBP1 silencing on melanoma stem cell proliferation, the cell cycle of PTBP1-silenced stem cells was examined with flow cytometry. The results showed that the depletion of PTBP1 significantly increased the proportion of cells in G0/G1 phase (Fig. 5C), indicating that the PTBP1 silencing led to the cell cycle arrest of melanoma stem cells. At the same time, the PTBP1 silencing significantly increased the percentage of apoptotic cells compared with the control (Fig. 5D). These data indicated that PTBP1 could promote the proliferation of melanoma stem cells by inducing cell cycle arrest and inhibiting apoptosis.

To explore the effects of PTBP1 knockdown on the stemness of melanoma stem cells, the PTBP1-silenced melanoma stem cells were subjected to the detection of stemness genes’ expressions. Quantitative real-time PCR results showed that the PTBP1 silencing led to significant downregulations of stemness genes (*Klf4*, *Nanog*, *Oct4*, and *Sox2*) compared with the controls (Fig. 5E), indicating that PTBP1 played a positive role in maintaining the stemness of melanoma stem cells. At the same time, the PTBP1 silencing significantly decreased the percentage of tumorsphere formation of melanoma stem cells compared with the control (Fig. 5F). These data indicated that PTBP1 was required for maintaining the stemness of melanoma stem cells.

In clinic, the analysis of the data from GEPIA database (http://gepia.cancer-pku.cn/index.html) showed that the PTBP1 expression in the solid tumors of melanoma patients was upregulated compared with that of the healthy donors (Fig. 5G). At the same time, the Kaplan–Meier survival analysis revealed that the melanoma patients with high PTBP1 expression level had distinct shorter survival rate compared with the patients with low PTBP1 level (Fig. 5H). These data demonstrated that the PTBP1 expression was positively associated with melanoma progression, indicating that PTBP1 might serve as a new target for melanoma therapy.

Collectively, our findings indicated that PTBP1 was required for maintaining the stemness of melanoma stem cells.

### Splicing of LHFPL3-AS1 mediated by PTBP1

**Fig. 6:**
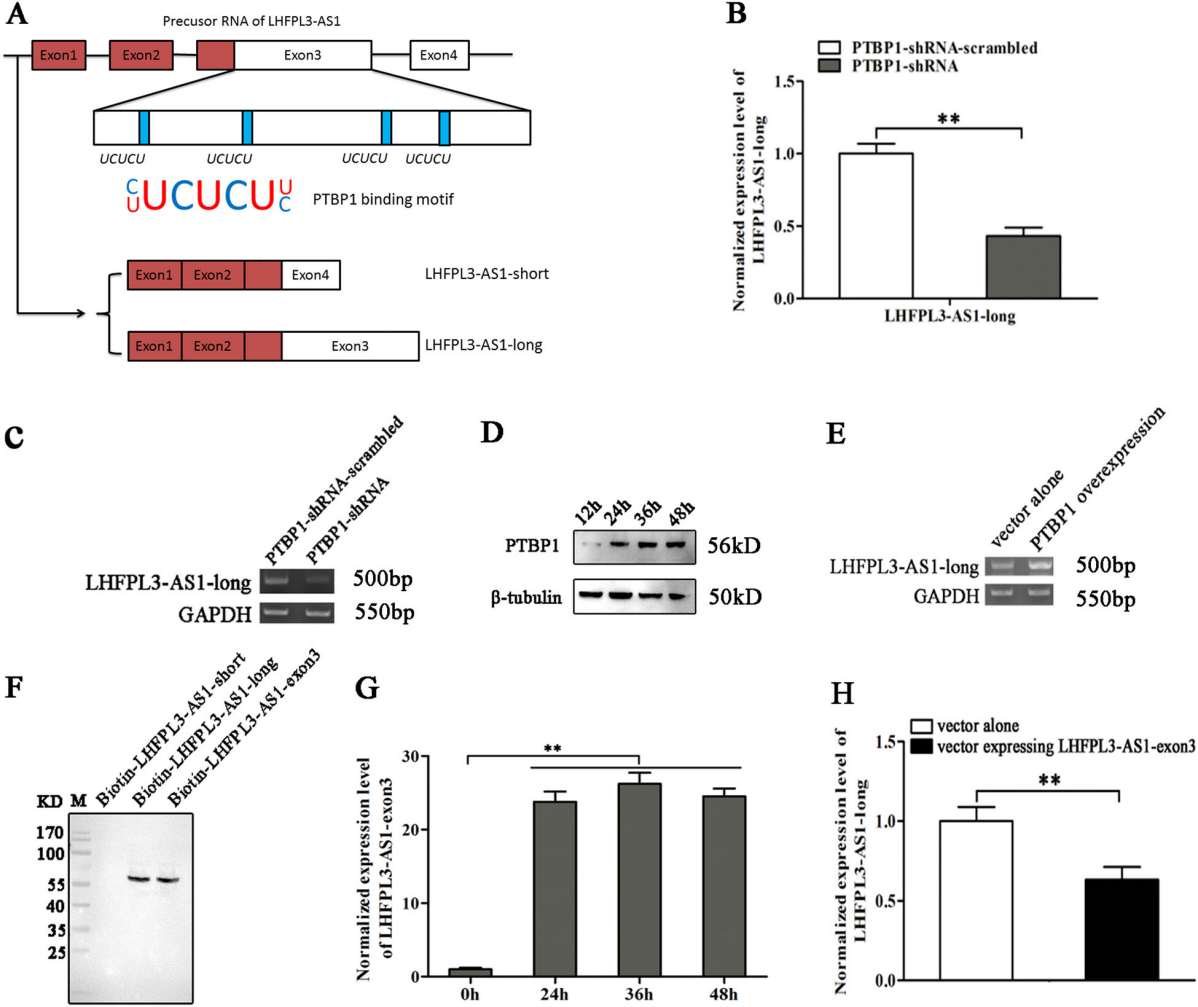
Splicing of LHFPL3-AS1 mediated by PTBP1. **A** Schematic diagram of the potential splicing of LHFPL3-AS1. The precursor RNA of LHFPL3-AS1 was spliced by PTBP1, possibly generating LHFPL3-AS1-long. **B** Impact of PTBP1 silencing on LHFPL3-AS1-long expression in melanoma stem cells. The LHFPL3-AS1-long expression level in cells with decreased PTBP1 was examined using quantitative real-time PCR (**p < 0.01). **C** Agarose gel electrophoresis of LHFPL3-AS1-long. LHFPL3-AS1-long in the PTBP1 stably silenced melanoma stem cells was amplified with RT-PCR and examined by agarose gel electrophoresis. **D** Overexpression of PTBP1 in melanoma stem cells. PTBP1 stably silenced cells were transfected with the recombinant plasmid expressing PTBP1. The expression level of PTBP1 was detected using Western blot. β-tubulin was used as a loading control. **E** Rescue effects of PTBP1 on the expression of LHFPL3-AS1-long. At 48 h after the transfection of the recombinant plasmid expressing PTBP1, LHFPL3-AS1-long was amplified using RT-PCR. **F** Detection of the protein bound to LHFPL3-AS1. The biotinylated LHFPL3-AS1 fragments were incubated with the lysate of melanoma stem cells, respectively. The RNA pull-down products were analyzed using Western blot with anti-PTBP1 IgG. M, protein marker. **G** Overexpression of LHFPL3-AS1-exon3 in melanoma stem cells. The expression level of LHFPL3-AS1-exon3 was investigated using quantitative real-time PCR at different time (**p < 0.01). **H** Impact of LHFPL3-AS1 exon3 on the splicing of LHFPL3-AS1 precursor. The LHFPL3-AS1-long level was examined using quantitative real-time PCR in the LHFPL3-AS1-exon3 overexpressed melanoma stem cells (**p < 0.01).

As reported, PTBP1 plays an important role in alternative splicing^21^. Therefore, the role of PTBP1 in the splicing of LHFPL3-AS1 was explored. The sequence analysis showed that there were several motifs (UCUCU) in the LHFPL3-AS1 precursor and LHFPL3-AS1-long (Fig. 6A), which could be bound by PTBP1 protein^20^, suggesting that the splicing of LHFPL3-AS1 regulated by PTBP1 contribute to the biogenesis of LHFPL3-AS1-long.

To reveal the influence of PTBP1 on the biogenesis of LHFPL3-AS1-long, PTBP1 was knocked down in melanoma stem cells, followed by the examination of LHFPL3-AS1 isoforms. The results showed that PTBP1 depletion significantly reduced the level of LHFPL3-AS1-long (Fig. 6B, C). At the same time, when PTBP1 was rescued in PTBP1 stable silenced cells (Fig. 6D), the expression level of LHFPL3-AS1-long was also recovered (Fig. 6E). These data demonstrated that PTBP1 was responsible for the biogenesis of LHFPL3-AS1-long in melanoma stem cells. To further confirm the role PTBP1 in the biogenesis of LHFPL3-AS1-long, RNA pull-down assays were conducted using biotin-labeled RNAs. The results demonstrated that the PTBP1 protein was bound to LHFPL3-AS1-long and LHFPL3-AS1-exon3, but not LHFPL3-AS1-short (Fig. 6F). To evaluate the influence of the exon3 of LHFPL3-AS1 on the PTBP1-mediated splicing of LHFPL3-AS1, the exon3 of LHFPL3-AS1 was overexpressed in melanoma stem cells (Fig. 6G). Quantitative real-time PCR showed that the exon3 overexpression decreased the LHFPL3-AS1-long level compared with the control (Fig. 6H), indicating the PTBP1 protein mediated the splicing of LHFPL3-AS1 by binding to its exon3. These results revealed that the PTBP1 protein was required for the biogenesis of LHFPL3-AS1-long in melanoma stem cells.

### Roles of PTBP1 and LHFPL3-AS1 in tumorigenesis of melanoma stem cells in vivo

**Fig. 7:**
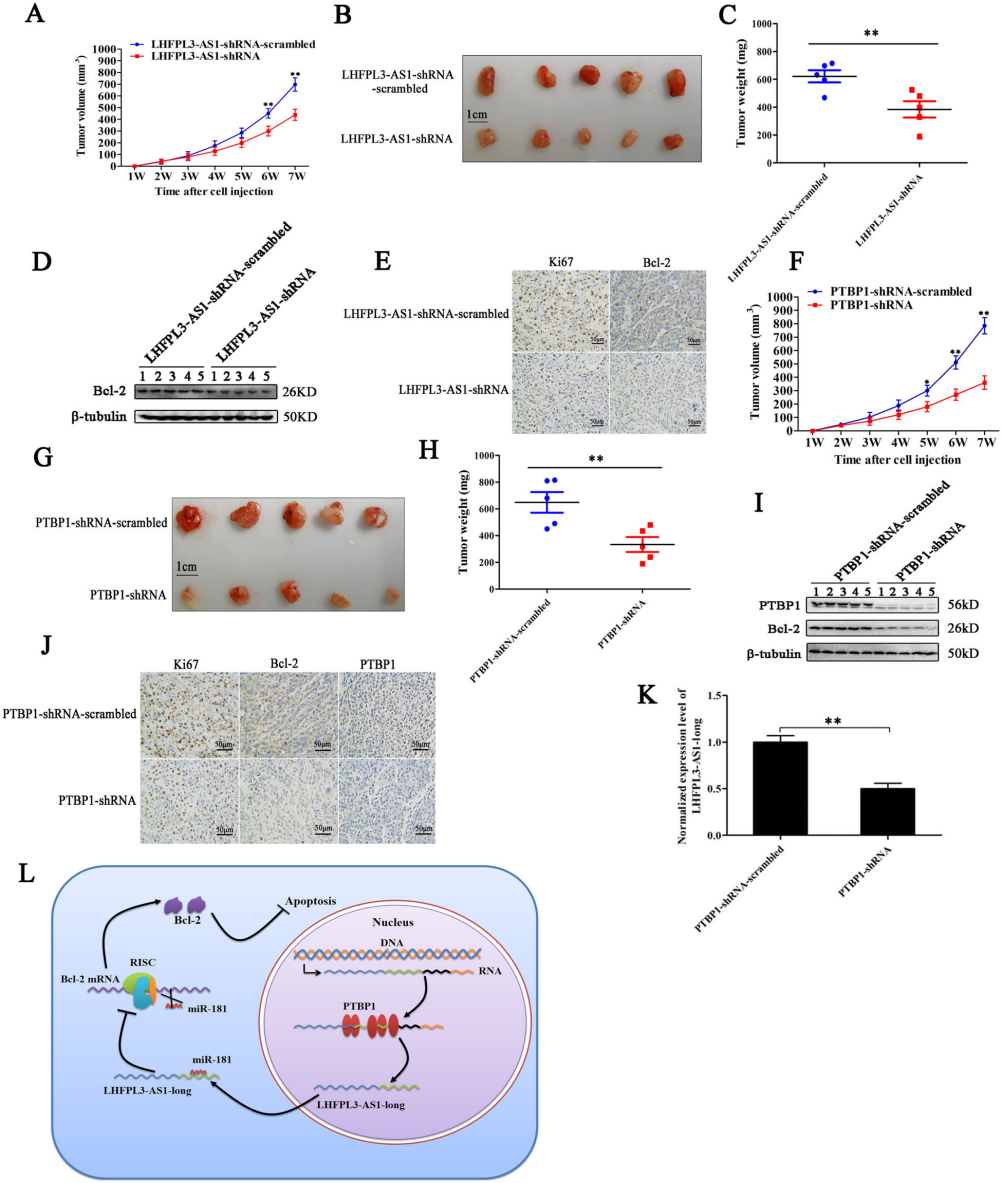
Roles of PTBP1 and LHFPL3-AS1 in tumorigenesis of melanoma stem cells in vivo. **A** Effects of LHFPL3-AS1 silencing on the tumor growth in mice. Melanoma stem cells treated with LHFPL3-AS1-shRNA were injected into nude mice. Then the tumor volume in mice was measured every week. Each point represented the mean of five mice (**p < 0.01). **B** Evaluation of solid tumor size derived from mice. Seven weeks after cell inoculation, the mice were sacrificed and the tumor size was examined. **C** Solid tumor weight isolated from mice (**p < 0.01). **D** Western blot analysis of Bcl-2 in xenografts. β-tubulin was used as loading control. **E** Immunohistochemical analysis of Ki67 and Bcl-2 in solid tumors. The shade of brown represented Ki67, Bcl-2 protein level. Scale bars, 50 µm. **F** Impact of PTBP1 silencing on solid tumor growth in mice. Melanoma stem cells transfected with PTBP1-shRNA or PTBP1-shRNA-scrambled were inoculated into nude mice. The tumor volumes of mice were examined every week. Each point represented the mean of five mice (*p < 0.05; **p < 0.01). **G** Evaluation of solid tumor size isolated from mice. Seven weeks after cell inoculation, the mice were sacrificed and the tumor sizes were examined. **H** Solid tumor weights of mice with different treatments (**p < 0.01). **I** Western blot analysis of PTBP1 and Bcl-2 proteins in solid tumors. β-tubulin was used as a control. **J** Immunohistochemical analysis of Bcl-2 and PTBP1 in solid tumors. The shade of brown represented Ki67, Bcl-2, and PTBP1 protein level. Scale bars, 50 µm. **K** Impact of PTBP1 silencing on the expression of LHFPL3-AS1-long in solid tumors. The expression level of LHFPL3-AS1-long was analyzed by quantitative real-time PCR (**p < 0.01). **L** Model for the role of PTBP1-LHFPL3-AS1-apoptosis pathway in tumorigenesis of melanoma stem cells.

In order to explore the role of LHFPL3-AS1 in tumor progression in vivo, the melanoma stem cells transfected with LHFPL3-AS1-shRNA were subcutaneously injected into nude mice, followed by monitoring tumor growth every week for 6 weeks. The results showed that the LHFPL3-AS1 depletion significantly inhibited tumorigenesis of melanoma stem cells in vivo compared with the control (Fig. 7A). The sizes and weights of xenografted solid tumors were much smaller and lower than those of the control (Fig. 7B, C). These data demonstrated that LHFPL3-AS1 played a positive role in tumorigenesis of melanoma stem cells in vivo. Western blots indicated that the Bcl-2 protein was significantly downregulated in LHFPL3-AS1-silenced xenografts, but not in the control group (Fig. 7D). The immunohistochemistry analysis yielded the similar results (Fig. 7E). These data indicated that LHFPL3-AS1 could promote tumorigenesis of melanoma stem cells in vivo.

To evaluate the role of PTBP1 in tumorigenesis of melanoma stem cells in vivo, the PTBP1-silenced melanoma stem cells were subcutaneously injected into nude mice, followed by the examination of tumor growth. The results demonstrated that the PTBP1 silencing significantly decreased the growth of tumors in mice compared with the control (Fig. 7F). At the same time, the volume and weight of solid tumors of the PTBP1-silenced cells were significantly reduced (Fig. 7G, H). Western blots and immunohistochemical analysis of xenografted tumors showed that the PTBP1 silencing significant downregulated the expression of Bcl-2 (Fig. 7I, J). The PTBP1 silencing also led to a significant downregulation of LHFPL3-AS1-long in solid tumors (Fig. 7K). These data indicated that PTBP1 played a positive role in tumorigenesis of melanoma stem cells in vivo.

Taken the above results together, the findings revealed that PTBP1 promoted the splicing of LHFPL3-AS1 precursor to generate LHFPL3-AS1-long, and subsequently LHFPL3-AS1-long inhibited the mRNA degradation of *Bcl-2* (the target gene of miR-181) by its direct interaction with miR-181a-5p to suppress apoptosis of melanoma stem cells, thus leading to tumorigenesis of melanoma stem cells in vivo (Fig. 7L).

## Discussion

Cancer stem cells often reprogram the expression profiles of a wide range of genes to support their self-renewal and differentiation, which have been considered as hallmarks of stem cells^14^. Over the last decade, lncRNAs have been proposed to play very important roles in the transcriptional regulations of genes^22^. However, the roles of lncRNAs in tumorigenesis of cancer stem cells are not extensively explored. In this study, the findings revealed that LHFPL3-AS1-long, one of two LHFPL3-AS1 isoforms, was required for maintaining the stemness of melanoma stem cells via directly interacting with miR-181 to inhibit the mRNA degradation of its target gene *Bcl-2* and apoptosis of melanoma stem cells. As reported, lncRNAs can function *in cis* or *in trans*^22^. For the roles of lncRNAs *in cis*, they usually depend on their sequences and the DNA elements which are transcribed to regulate the expressions of neighboring genes. It is found that *LncTCF7* is required for the self-renewal and tumor propagation of liver cancer stem cells, which functions based on the recruitment of SWI/SNF complex to the neighboring promoter of TCF7 to regulate its expression^13^. The lncRNAs *in trans* are usually mediated by the interactions with the specific proteins and RNAs (ceRNAs) to regulate the behaviors of protein binders and other RNA molecules^23^. Characterizing the epigenetic landscape of genes encoding lncRNAs identifies a lncRNA *EPIC1*, which promotes cancer cell-cycle progression by interacting with MYC, thus enhancing MYC occupancy on target genes^23^. Up to date, numerous experimental evidences have shown that the competition for miRNAs plays an integral part in the regulation of both lncRNAs and mRNAs^8,24^. Although the diverse functions of lncRNA have been characterized, the influence of lncRNAs on the stemness of cancer stem cells has still poorly understood. Therefore, our findings provided novel insights into the role of lncRNAs in tumorigenesis of cancer stem cells.

Alternative pre-mRNA splicing is emerging as an important mechanism of genetic diversity, enabling a single gene to produce multiple RNA variants and distinct protein isoforms from an apparently limited number of loci in the genome^25^. As a critical mechanism during cell development and differentiation, alternative splicing is involved in tumor progression^26^. Whole-exome RNA sequencing data demonstrate that tumors have up to 30% more alternative splicing events than normal cells^27^, suggesting that aberrant alternative splicing can be one of the major molecular hallmarks of human cancers^28^. So far, however, the roles of splicing in the biogenesis of lncRNAs and its influence on tumorigenesis of cancer stem cells have not been well investigated. In this study, our results indicated that the PTBP1 protein was responsible for the biogenesis of LHFPL3-AS1-long by promoting the splicing of LHFPL3-AS1 precursor in melanoma stem cells. As reported, alternative splicing is regulated by an extensive protein–RNA interaction network involving *cis* elements within the pre-mRNA and *trans*-acting factors that bind to these cis elements^21^. Generally the splicing factors include serine/arginine-rich proteins and heterogeneous nuclear ribonucleoproteins (hnRNPs) that positively or negatively control splicing in different pre-mRNA regions^29^. PTBP1 (also termed as hnRNP I), a member of hnRNPs, is a well-known suppressive regulator of alternative splicing by inhibiting intron and exon definition^30,31^. Our findings showed that PTBP1 specifically interacted with the motif (UCUCU) in exon3 of in the LHFPL3-AS1 precursor. The silencing of LHFPL3-AS1 or PTBP1 could suppress, but not completely block, tumor progression, which is a universal phenomenon for RNAi-dependent cancer therapy^23^. Even if an oncogene is knocked out in cancer cells, the oncogene knockout can’t block tumorigenesis, owing to the genetic redundancy and functional compensation through some buffering mechanisms^32^. In this context, our study revealed a mechanistic crosstalk among an onco-splicing factor, alternative splicing event of lncRNA and tumorigenesis of melanoma stem cells, enabling PTBP1 and the splicing of LHFPL3-AS1 to serve as the attractive therapeutic targets for melanoma.
